# Detection of significantly high vitreous concentrations of fatty acid-binding protein 4 in patients with proliferative diabetic retinopathy

**DOI:** 10.1038/s41598-021-91857-1

**Published:** 2021-06-11

**Authors:** Kaku Itoh, Masato Furuhashi, Yosuke Ida, Hiroshi Ohguro, Megumi Watanabe, Soma Suzuki, Fumihito Hikage

**Affiliations:** 1 Department of Ophthalmology, Sapporo Medical University School of Medicine, Sapporo, Japan; 2 Department of Cardiovascular, Renal and Metabolic Medicine, Sapporo Medical University School of Medicine, Sapporo, Japan

**Keywords:** Retinal diseases, Diabetes complications

## Abstract

The fatty acid-binding protein4 (FABP4) and vascular endothelial growth factor A (VEGFA) play key roles in the metabolic and cardiovascular diseases, and proliferative diabetic retinopathy (PDR), respectively. To identify FABP4 in vitreous fluid in PDR, vitreous concentrations of FABP4 (V-FABP4) and VEGFA (V-VEGFA) from PDR (n = 20) and non-PDR (n = 20) patients were determined by Enzyme-Linked ImmunoSorbent Assays. The data, which included height and weight, systemic blood pressures, several blood biochemical parameters and blood flow at the optic nerve head (ONH) by laser speckle flowgraphy (LSFG) were collected. The levels of V-FABP4 and V-VEGFA were significantly higher in PDR patients than in non-PDR patients (P < 0.001) with a high positive correlation (r = 0.72, P < 0.001) between them. The findings were not affected by body mass index values and the presence of vitreous hemorrhaging. Among the clinical parameters, V-FABP4 correlated positively with creatinine and negatively with age and aspartate transaminase (AST) levels, while V-VEGFA correlated positively with fasting plasma glucose and hemoglobin A1c (HbA1c) levels but negatively with AST. Multiple regression analyses indicated that V-VEGFA, or V-FABP4, AST and HbA1c were independent predictors of V-FABP4 or V-VEGFA, respectively. Both were negatively correlated, but more evident in V-FABP4, with the ONH ocular blood flow.

## Introduction

Diabetic retinopathy (DR) is a grave vision-threatening retinal complication of diabetes mellitus (DM), remains one of the leading causes of blindness especially among the working age population worldwide. Proliferative diabetic retinopathy (PDR), a progressive and serious stage of DR due to retinal ischemia, is characterized by neovascularization (NV), vitreous hemorrhaging (VH) and tractional retinal detachment (TRD), all of which are major causes of blindness in patients with DM^1^. In terms of the molecular pathology of DR, vascular endothelial growth factor (VEGF) acts as a pivot factor in the development of neovascularization^2^. Previous studies have revealed that the intra-vitreous administration of anti-VEGF agents as an adjunctive therapy before vitreous surgery caused a significant decrease in intraoperative bleeding, shortening the surgical time as well as consequently improving surgical outcome^3^. Although anti-VEGF therapy is well recognized as a powerful and beneficial therapeutic strategy, its efficacy is transient and anti-VEGF mono-therapy typically does not stop the disease progression of DR^4,5^. It therefore appears that additional independent factors, other than VEGF mediated mechanisms, must be present in terms of the molecular etiology of PDR.

Fatty acid-binding proteins (FABPs) that act as intracellular lipid chaperones are a group of molecules that coordinate lipid responses in cells^6,7^. FABPs are abundantly expressed 14–15-kDa proteins that can reversibly bind hydrophobic ligands such as long-chain, saturated and unsaturated fatty acids with high affinity^6,7^. It has been postulated that FABPs facilitate the transport of lipids to specific compartments within the cell, such as the endoplasmic reticulum where they are involved in signaling, trafficking, and membrane synthesis, to mitochondria or peroxisomes for oxidation, to cytosolic or other enzymes to regulate their activity, to the nucleus for lipid-mediated transcriptional regulation, and to lipid droplets for storage^6,7^. Among the currently known FABPs, the FABP4, known as adipocyte FABP (A-FABP) or aP2, is expressed in both adipocytes and macrophages, and can be detected in most bodily fluids. Elevated serum concentrations of FABP4 are associated with obesity^8^, insulin resistance^9^, hypertension (HT)^10^, dyslipidemia^11^, atherosclerosis^12^, renal dysfunction^13^, purine metabolism^14^, heart failure and cardiovascular events^15^. Recent studies have also demonstrated that the concentration of FABP4 can be modulated by administering therapeutic drugs for HT, dyslipidemia and DM^9^. These collective observations suggest that FABP4 could also be rationally involved in ocular pathophysiology, especially in DM induced retinopathy. Although another FABP member, FABB5 has been detected within lens so far^16^, our knowledge of the extent of involvement of FABP4 within DR is currently very limited.

In the current study, to elucidate the pathological involvement of FABP4 within the PDR, we surgically collected vitreous specimens from patients with PDR or non-PDR (epiretinal membranes or macular holes) and measured the FABP4 and VEGF concentrations in these samples.

## Methods

This study conformed to the principles outlined in the Declaration of Helsinki and was performed with the approval of the institutional ethical committee of our institution. Written informed consent was received from all of the participating subjects.

### Patients

Twenty patients who had been consecutively operated on (n = 20 eyes) with PDR (mean age 63.27 ± 11.90 years; 10 male and 10 female, vitreous hemorrhage; 12 eyes, traction retinal detachment; 7 eyes, neovascular glaucoma; 5 eyes, maculopathy; 3 eyes) and 20 patients (mean age 69.2 ± 9.1 years; 8 male and 12 female) with a macular hole (n = 7 eyes) or an epiretinal membrane (n = 13 eyes) requiring vitrectomy were recruited from the Muroran municipal hospital during Jan to Dec, 2017. The 20 former and 20 latter patients were categorized as PDR group and non-PDR group, respectively. In order to determine a suitable surgical indication of vitrectomy, all patients underwent a complete ophthalmologic evaluation before surgery with a best-corrected visual acuity (BCVA) determination, slit-lamp examination, fundus examination, intraocular pressure measurement, gonioscopy, and optical coherence tomography. A clinical preoperative and intraoperative assessment of disease activity was performed by one experienced retina specialist (K.I). In all patients, under systemic anesthesia, 25 or 27-gauge three-port pars plana vitrectomies were performed (Alcon Constellation Vision System), and simultaneous cataract surgery was added except for 2 eyes each from 20 PDR and 20 non-PDR patient groups. Inter limiting membrane pealing, or air or 10–20% SF6 gas tamponade was performed for 10 PDR eyes and 14 for non-PRD eyes, or 17 PDR eyes and 19 non-PDR eyes, respectively during the surgery. In 12 out of 20 eyes from PDR patients, vitreous hemorrhaging was confirmed prior to the surgery. Post-operatively, no serious complications except for slight vitreous hemorrhaging were observed and none of the eyes have required reoperations as of this writing. Data regarding each patient’s general conditions and diabetes control were obtained from the patient and from the patient’s general practitioner or diabetologist.

Body height and weight measurements, blood pressure measurements and the collection of peripheral blood specimens for a complete blood count and biochemical analyses were performed as described previously^17^.

### Biochemistry measurements

Biochemistry measurements of the vitreous concentrations of FABP4 (V-FABP4) or VEGFA (V-VEGFA) and several blood chemistry analyses including plasma glucose levels, hemoglobin A1c (HbA1c), creatinine (Cr), blood urea nitrogen (BUN), uric acid, aspartate transaminase (AST), alanine aminotransferase (ALT), γ-glutamyl transpeptidase (γ-GTP), lipid profiles, including total cholesterol and triglycerides, high-sensitivity C-reactive protein (hsCRP) and estimated glomerular filtration rate (eGFR) were performed as described recently^17^. Briefly, undiluted vitreous specimens from 20 PDR and 20 non-PDR subjects were collected during their initial the core vitrectomy to pay an extreme care to avoid contamination of extraocular blood. The concentrations of V-FABP4 (ng/mg protein) or V-VEGFA (pg/mg protein) were determined by enzyme-linked immunosorbent assay for FABP4 (Biovendor R&D, Modrice, Czech Republic) or human VEGFA (Fuji film Wako. Co., Japan), respectively after adjustment by vitreous protein concentrations analysis (Pierce BCA Protein Assay Kit, Pierce Biotechnology, Rockford USA).

### Laser speckle flowgraphy (LSFG)

The images of the speckle contrast pattern produced by interference as the laser beam was scattered by erythrocytes moving through the ocular fundus vessels were obtained by a fundus camera equipped with an 830 nm diode laser and a charge-coupled device sensor (750 × 360 pixels) (LSFG-NAVI; Softcare Co, Ltd., Fukuoka, Japan) as described previously^18,19^. The LSFG images that were acquired were continuously monitored at 30 frames/sec over a 4-s period and averaged to produce a composite map of ocular blood flow. As a demonstrable indicator of the ocular blood flow at a specific site, the mean blur rate (MBR), as arbitrary units (AU), were calculated and the values at several sites were reconstituted to form a 2-dimensional color-coded map of blood flow velocity. In the current study, we investigated four MBR categories; (1) Average; overall of the optic nerve head (ONH), (2) the vascular area of the ONH (MV) including effects of choroidal vessels, (3) the tissue area of the ONH (MT), and (4) MV-MT (to exclude the effects of choroidal vessels from the MV). All measurements were performed in triplicate and the mean MBR value was calculated. Eye positions were continuously monitored during the LSFG analysis with an auto tracking function, to confirm that the same area was captured again during subsequent examinations.

### Statistical analysis

Means ± SD for normal distributions or medians (interquartile ranges) for skewed variables were used for expressions of numeric variables. Statistical analyses including (1) intergroup differences by the chi-square test, (2) comparison between two groups by the Mann–Whitney's U test, (3) the distribution of each parameter for its normality using the Shapiro–Wilk W test, (4) logarithmically transformation of non-normally distributed parameters for regression analyses, (5) correlations between two continuous variables by Pearson’s correlation coefficient, and (6) multivariable linear regression models analysis to explore independent parameters of V-FABP4 and V-VEGFA were performed using JMP 14.3.0 for Macintosh (SAS Institute, Cary, NC) as described recently^17^. Age, sex and variables with significant correlations determined by Pearson’s coefficient were incorporated in the multivariable models after consideration of multicollinearity. The relationships were expressed with standardized regression coefficient (β). A p value of < 0.05 was considered statistically significant.

## Results

### Characteristics of patient background

Characteristics of the enrolled patients’ backgrounds (PDR; n = 20, non-PDR; n = 20) are shown in Table 1. In a comparison of the PDR and non-PDR patient groups, fasting glucose levels, HbA1c and blood urea nitrogen, or mean age, AST and ALT levels were significantly higher or lower, respectively, in PDR group. No significant difference was observed with respect to sex, body mass index (BMI), systolic and diastolic blood pressures, total cholesterol, triglycerides, Cr, eGFR, uric acid, γGTP and hsCRP between groups. In terms of ocular blood flow at the ONH, all LSFG indexes including MA, MV, MT and MV-MT were significantly lower in the PDR group as compared to the non-PDR group.

**Table 1:** Characteristics of the patients (n = 40).

	All	non-PDR	PDR	P
n	40	20	20	
Sex (Male/female)	18/22	8/12	10/10	0.525
Age (years)	66 ± 9	69 ± 9	63 ± 9	0.019
Body mass index	23.6 ± 3.7	23.1 ± 3.2	24.0 ± 4.3	0.453
Systolic blood presure (mmHg)	138 ± 21	138 ± 16	137 ± 25	0.953
Diastolic blood pressure (mmHg)	77 ± 11	79 ± 11	75 ± 12	0.334
**Biochemical data**				
AST (IU/L)	21 (16–27)	26 (20–30)	17 (14–22)	< 0.001
ALT (IU/L)	20 (14–27)	25 (16–30)	16 (11–21)	0.014
γGTP (IU/L)	24 (15–51)	28 (15–65)	21 (15–45)	0.330
Blood urea nitrogen (mg/dL)	18 ± 9	15 ± 4	21 ± 12	0.031
Creatinine (mg/dL)	0.7 (0.6–0.9)	0.7 (0.6–0.8)	0.8 (0.6–1.0)	0.198
eGFR (mL/min/1.73m^2^)	67.0 ± 25.7	70.6 ± 16.5	63.3 ± 32.5	0.378
Uric acid (mg/dL)	5.4 ± 1.2	5.2 ± 1.2	5.6 ± 1.3	0.309
Total choleterol (mg/dL)	199 ± 42	203 ± 41	195 ± 43	0.514
Triglycerides (mg/dL)	148 (103–221)	120 (95–222)	157 (136–214)	0.148
Fasting glucose (mg/dL)	141 (105–171)	120 (102–153)	167 (140–184)	0.042
Hemoglobin A1c (%)	6.5 ± 1.0	6.1 ± 0.8	6.9 ± 1.1	0.010
hsCRP (mg/dL)	0.05 (0.04–0.12)	0.06 (0.04–0.12)	0.05 (0.03–0.12)	0.714
**Laser speckle flowgraphy**	[n = 33]	[n = 20]	[n = 13]	
MA	19.3 ± 7.4	22.6 ± 6.5	14.3 ± 5.7	0.001
MV	33.4 ± 13.1	39.1 ± 10.8	24.6 ± 11.5	0.001
MT	11.5 ± 3.3	12.5 ± 2.9	9.9 ± 3.2	0.022
MV-MT	21.9 ± 11.0	26.6 ± 9.2	14.7 ± 10.0	0.001

Variables are expressed as number, means ± SD or medians (interquartile ranges).

*AST* aspartate transaminase; *ALT* alanine transaminase; *eGFR* estimated glomerular filtration rate; *γGTP* γ-glutamyl transpeptidase; *hsCRP* high-sensitivity C-reactive protein; *MA* mean blur rate of all optic verve head area; *MT* mean blur rate of tissue area of the optic nerve head; *MV* mean blur rate of vascular area of the optic nerve head; *PDR* proliferative diabetic retinopathy.

### V-FABP4 and V-VEGFA concentrations between PDR and non-PDR patients

**Figure 1:**
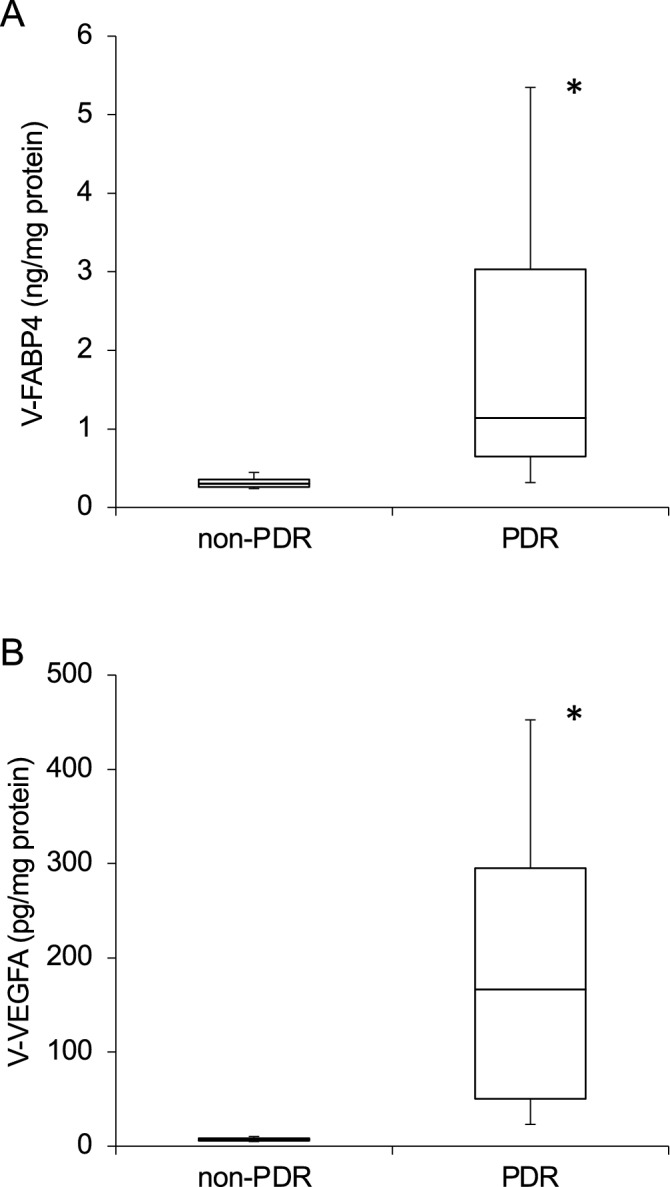
Levels of vitreous FABP4 (V-FABP4) and VEGFA (V-VEGFA) in patients with PDR and non-PDR. Undiluted vitreous specimens obtained surgically from patients with PDR (n = 20) and non-PDR (n = 20) were subjected to Enzyme-Linked Immuno-Sorbent Assay (ELISA) for FABP4 and VEGFA. The levels of V-FABP4 (ng/mg protein) and V-VEGFA (pg/mg protein) in both groups were plotted in panel A and B, respectively. *FABP4* fatty acid-binding protein 4; *VEGFA* vascular endothelial growth factor A; *V-FABP4* vitreous FABP4; *V-VEGFA* vitreous VEGFA. *P < 0.001 vs. non-PDR.

**Figure 2:**
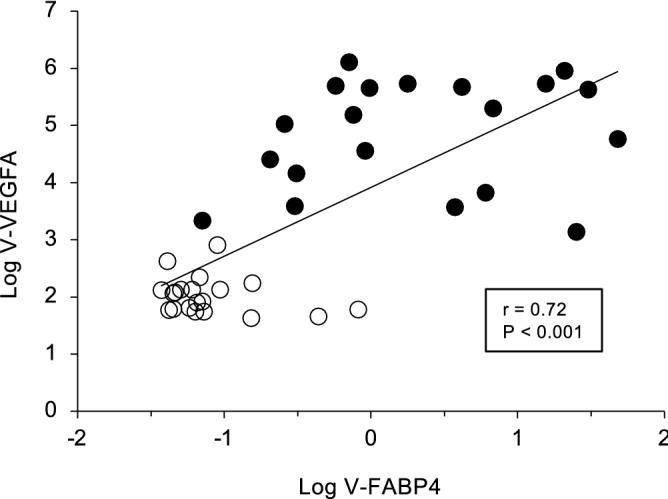
Correlations between Log V-FABP4 and Log V-VEGFA. Levels of Log V-FABP4 were plotted against Log V-VEGFA for each subject panel A, n = 40, r = 0.72, P < 0.001). Open circles, subjects with non-PDR; closed circles, subjects with PDR; *FABP4* fatty acid-binding protein 4; *VEGFA* vascular endothelial growth factor A; *V-FABP4* vitreous FABP4; *V-VEGFA* vitreous VEGFA.

As shown in Fig. 1, the concentrations of both V-FABP4 and V-VEGFA were significantly elevated in patients with PDR compared with those with non-PDR (P < 0.001). In addition, an extremely high positive correlation (r = 0.72, P < 0.001) was observed between the concentrations of V-FABP4 and V-VEGFA (Fig. 2). It is known that FABP4 is produced by adipocytes as well as macrophages, and is secreted into several bodily fluids including the peripheral blood^20^. Since fat tissue is generally more abundant in females than in males as well as in higher BMI subjects, serum FABP4 levels are generally higher in females than males, as well as in higher BMI subjects^8,9^.

### Correlation analysis of several factors including sex, BMI values, the presence of vitreous hemorrhaging, and the presence of DM and/or HT with V-FABP4 and V-VEGFA

If V-FABP4 were derived from peripheral blood, those levels might be significantly affected by differences in sex, BMI values, and the presence of vitreous hemorrhaging. However, BMI values were not significantly different between the PDR and non-PDR patients groups (Table 1), levels of V-FABP4 (male [n = 18] vs. female [n = 22]: 0.58 [0.30–1.29] vs. 0.44 [0.30–1.41] ng/mg protein, P = 0.78) and V-VEGFA (male [n = 18] vs. female [n = 22]: 40.9 [8.3–160.0] vs. 9.9 [6.5–293.0] pg/mg protein, P = 0.63) were comparable between sex among all patients (n = 40), and the levels of both V-FABP4 and V-VEGFA were not affected irrespective of whether vitreous hemorrhaging was detected in PDR patients (Table 2). In addition, among the non-PDR patient group, V-FABP4 (male [n = 8] vs. female [n = 12]: 0.29 [0.25–0.35] vs. 0.30 [0.28–0.41] ng/mg protein, P = 0.67), BMI (male [n = 8] vs. female [n = 12]: 23.8 ± 2.4 vs. 22.7 ± 3.7, P = 0.37) and V-VEGFA (male [n = 8] vs. female [n = 12]: 8.2 [6.0–12.6] vs. 6.8 [5.7–8.4] pg/mg protein, P = 0.40) were also comparable between genders. Furthermore, it is also known that serum FABP4 levels are elevated by insulin resistance and atherosclerosis, which causes DM and hypertension (HT), respectively. As shown in Table 2, V-FABP4 concentrations were not significantly altered by association with DM among patients with non-PDR and with HT among patients with PDR, although significantly higher V-FABP4 concentrations were observed in the non-PDR patients with HT. In contrast, V-VEGFA concentrations were not significantly altered by association with DM in patients with non-PDR and with HT in patients with PDR or non-PDR (Table 2). These collective results suggest that the origin of V-FABP4 in PDR may be within eyes rather than the peripheral blood circulation, similar to V-VEGFA.

**Table 2:** Comparisons of V-FABP4 and V-VEGFA in comorbidity.

	non-PDR (n = 20)			PDR (n = 20)		
	(−)	(+)	P	(−)	(+)	P
**Vitreous hemorrhage**						
n	20	0		9	11	
V-FABP4 (ng/mg protein)	0.30 (0.26–0.35)	–	− 1.76 (0.69–3.50)	0.99 (0.60–2.29)	0.939	
V-VEGFA (pg/mg/protein)	7.4 (5.9–8.4)	–	− 95.1 (32.0–302.1)	179.4 (82.6–287.1)	0.518	
**Diabetes mellitus**						
n	16	4		0	20	
V-FABP4 (ng/mg protein)	0.30 (0.26–0.34)	0.34 (0.27–0.61)	0.422	–	1.14 (0.65–3.03)	–
V-VEGFA (pg/mg protein)	7.4 (6.0–8.4)	7.1 (5.4–12.6)	0.850	–	166.4 (50.3–295.1)	–
**Hypertension**						
n	12	8		12	8	
V-FABP4 (ng/mg protein)	0.28 (0.25–0.31)	0.34 (0.30–0.63)	0.019	0.91 (0.60–3.37)	1.57 (0.91–3.00)	0.418
V-VEGFA (pg/mg/protein)	7.9 (5.9–9.9)	6.5 (5.7–8.4)	0.589	88.9 (35.8–291.5)	233.3 (126.1–303.2)	0.263

Variables are expressed as number or medians (interquartile ranges).

*PDR* proliferative diabetic retinopathy; *V-FABP4* fatty acid-binding protein 4 in vitreous humor; *V-VEGFA* vascular endothelial growth factor A in vitreous humor.

### Correlation analysis of several peripheral blood parameters and ocular blood circulation indexes with V-FABP4 and V-VEGFA

**Figure 3:**
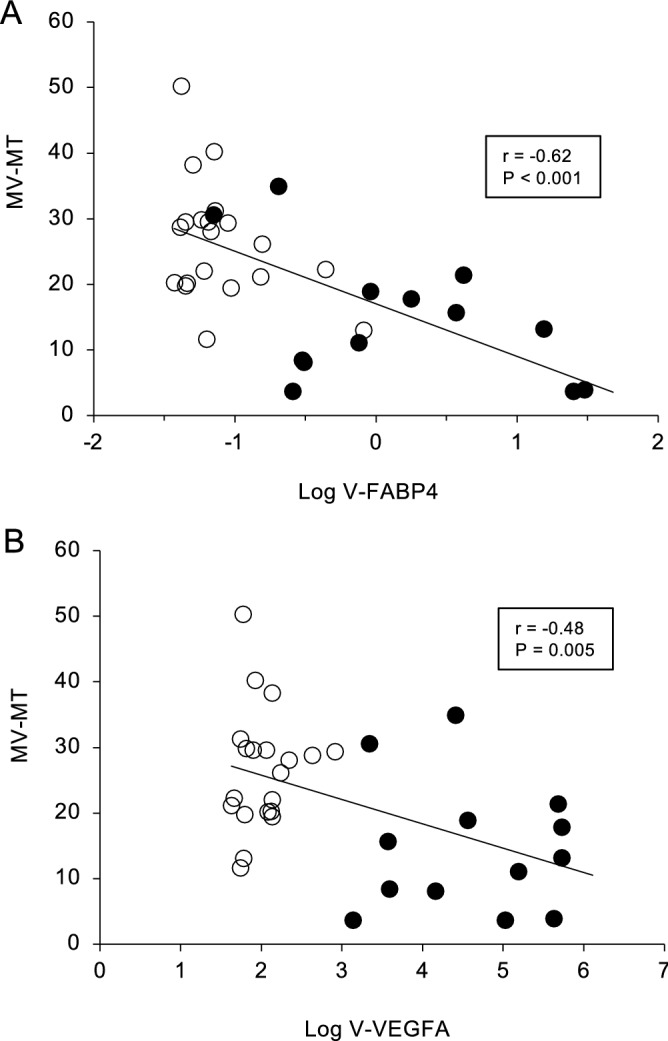
Correlations between Log V-FABP4 or Log V-VEGFA and ocular blood flow (MV-MT at baseline). Levels of Log V-FABP4 or Log V-VEGFA were plotted against MV-MT at the baseline for each subject (n = 40, panel A; Log V-FABP4; r = − 0.62, P < 0.001, panel B; Log V-VEGFA; r = − 0.48, P = 0.005). Open circles, subjects with non-PDR; closed circles, subjects with PDR; *FABP4* fatty acid-binding protein 4; *VEGFA* vascular endothelial growth factor A; *V-FABP4* vitreous FABP4; *V-VEGFA* vitreous VEGFA, *MBR* mean blur rate; *ONH* optic nerve head; *MV* the MBR of the vascular area of the ONH, *MT* the MBR of the tissue area of the ONH.

**Figure 4:**
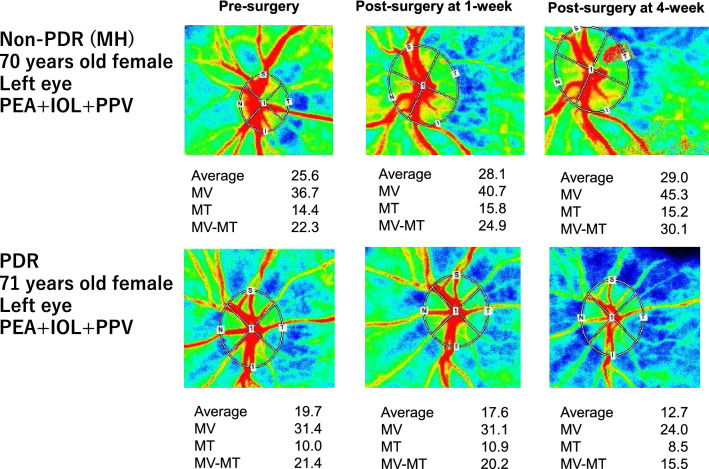
Representative LSFG images of non-PDR and PDR. Representative LSFG images of non-PDR patient (70 years old female, macular hole) and PDR patient (71 years old female) at pre-surgery and post-surgery 1- and 4-week are shown. Left eyes of both patients were treated by PEA (phacoemulsification), IOL (intraocular lens implantation) and PPV (pars plana vitrectomy). Average; the mean blur rate (MBR) of optic nerve head (ONH, designated by a circle), *MV* the MBR of the vascular area of the ONH, *MT* the MBR of the tissue area of the ONH, *I* inferior, *N* nasal, *S* superior, *T* temporal.

To elucidate the pathological significance of V-FABP4 toward PDR, correlations of the levels of V-FABP4 or V-VEGFA with several clinical parameters were further investigated. As shown in Table 3, Log V-FABP4 was positively correlated with Log V-VEGF (r = 0.72, P < 0.001) and Log Cr (r = 0.32, P = 0.041), and negatively correlated with mean age (r = − 0.36, P = 0.022) and Log AST (r = − 0.35, P = 0.026). While in contrast, Log VEGFA was positively correlated with Log V-FABP4 (r = 0.72, P < 0.001), Log fasting glucose levels (r = 0.35, P = 0.026) and Log HbA1c (r = 0.45, P = 0.004), and negatively correlated with Log AST (r = − 0.48, P = 0.002). Since VEGFA is known to be closely associated with the development of diabetic microangiopathy, such a positive correlation of Log V-VEGFA with Log fasting glucose levels or Log HbA1c is understandable. While in contrast, although a negative correlation with Log AST was observed in both Log V-FABP4 and Log V-VEGFA, Log V-FABP4 was correlated with different parameters, age (negatively) and Log Cr (positively), as Log V-VEGFA. In addition, negative correlations of both Log V-FABP4 and Log V-VEGFA with the LSFG ocular blood flow parameters at the optic nerve head (MA, MV and MV-MT) were observed, but such correlations were more evident in the case of V-FABP4 (Table 4 and Figs. 3 and 4). These data strongly suggest that fluctuations in V-FABP4 levels may be exclusively independent of V-VEGFA, but both could significantly affect the ocular blood circulation.

**Table 3:** Corelation anlyses for Log V-FABP4 and Log V-VEGFA (n = 40).

	Log V-FABP4		Log V-VEGFA	
	r	P	r	P
Age	− 0.36	0.022	− 0.29	0.065
Log V-VEGF	0.72	< 0.001	–	–
Log V-FABP4	–	–	0.72	< 0.001
Body mass index	0.16	0.324	0.05	0.763
Systolic blood pressure	0.04	0.812	0.01	0.944
Diastolic blood pressure	− 0.19	0.239	− 0.12	0.465
Log AST	− 0.35	0.026	− 0.48	0.002
Log ALT	− 0.20	0.216	− 0.31	0.052
Log γGTP	− 0.14	0.402	− 0.19	0.246
BUN	0.23	0.146	0.23	0.154
Log creatinine	0.32	0.041	0.17	0.286
eGFR	− 0.23	0.145	− 0.05	0.771
Uric acid	0.07	0.650	0.16	0.336
Total cholesterol	− 0.02	0.911	− 0.08	0.638
Log triglycerides	0.27	0.089	0.28	0.075
Log fasting glucose	0.23	0.157	0.35	0.026
Hemoglobin A1c	0.22	0.164	0.45	0.004
Log hsCRP	0.14	0.403	0.11	0.489

*AST* aspartate transaminase; *ALT* alanine transaminase; *eGFR* estimated glomerular filtration rate; *γGTP* γ-glutamyl transpeptidase; *hsCRP* high-sensitivity C-reactive protein; *V-FABP4* fatty acid-binding protein 4 in vitreous humor; *V-VEGFA* vascular endothelial growth factor A in vitreous humor.

**Table 4:** Correlation analyses for Log V-FABP4 and Log V-VEGFA with blood flow.

	Log V-FABP4		Log V-VEGFA	
	r	P	r	P
MA	− 0.53	0.001	− 0.50	0.003
MV	− 0.59	< 0.001	− 0.49	0.004
MT	− 0.27	0.129	− 0.34	0.054
MV-MT	− 0.62	< 0.001	− 0.48	0.005

*MA* mean blur rate of all optic verve head area; *MT* mean blur rate of tissue area of the optic nerve head; *MV* mean blur rate of vascular area of the optic nerve head; *V-FABP4* fatty acid-binding protein 4 in vitreous humor; *V-VEGFA* vascular endothelial growth factor A in vitreous humor.

### Stepwise multivariable regression analyses

To further study this, stepwise multivariable regression analyses were assessed using the correlated parameters as possible determinants, as shown in Tables 3 and 4. The data shown in Table 5 demonstrate that Log V-VEGFA, sex and MV-MT, or Log V-FABP4, Log AST and HbA1c were independent predictors of Log V-FABP4 or Log V-VEGFA, respectively, suggesting that V-FABP4 and V-VEGFA independently affect one another.

**Table 5:** Stepwise multivariable regression analyses for Log V-FABP4 and Log V-VEGFA.

	Log V-FABP4			Log V-VEGFA	
	β	P		β	P
Age	− 0.05	0.682	Age	− 0.07	0.481
Sex (male)	− 0.28	0.021	Sex (male)	0.01	0.884
Log V-VEGFA	0.52	< 0.001	Log V-FABP4	0.48	< 0.001
MV-MT	− 0.45	0.001	Log AST	− 0.36	0.001
			Hemoglobin A1c	0.37	0.001

*AST* aspartate transaminase; *MT* mean blur rate of tissue area of the optic nerve head; *MV* mean blur rate of vascular area of the optic nerve head; *V-FABP4* fatty acid-binding protein 4 in vitreous humor; *VVEGFA* vascular endothelial growth factor A in vitreous humor.

## Discussion

The mechanism responsible for the pathogenesis of DR is known to be extremely complex and involves numerous biochemical and inflammatory processes that are initiated upon long-term exposure to hyperglycemia^21^. During the development of DR, vascular endothelial dysfunction, pericyte loss, and neurodegeneration are simultaneously involved, and ultimately leads to the development of hypoxia and neovascularization^21^. During these progression processes, the local accumulation of cytokines, such as VEGF, tumor necrosis factor-alpha (TNF-⍺), and inducible nitric oxide synthase (iNOS) are substantially induced upon hypoxia in the diabetic retina^22^. This leads also to the accumulation of chemokines and adhesion molecules such as the intercellular adhesion molecule-1 (ICAM-1), and this, in turn, causes the migration of leukocytes towards the retinal endothelium, increased vascular permeability, and the breakdown of the blood–retinal barrier (BRB)^23^. This VEGF related signaling is pivotally involved in the pathogenesis of DR and therefore anti-VEGF therapy becomes an important therapeutic strategy^24^, in addition to classical treatments such as the administration of anti-angiogenic agents^25^ or corticosteroids intravitreal injections and laser therapy^26^. However, because of the complexity of the metabolic pathways that are activated during DR, as alluded to above, these single or combined therapies have only limited success^24–26^, and therefore additional therapeutic targets independent to the VEGF signaling need to be identified. In the current study, we found that the concentrations of both V-FABP4 and V-VEGFA were substantially elevated in eyes with PDR, and a significantly higher positive correlation (r = 0.72, P < 0.001) was observed between them. However, correlation analyses and stepwise multiple regression analyses for V-FABP and V-VEGFA strongly suggested that both factors were independently regulated, suggesting that V-FABP4 might also be involved in the pathogenesis of PDR.

The FABP4, primarily regarded as an adipocyte- and macrophage-specific protein, plays an important role in maintaining glucose and lipid homeostasis^8,15^. The issue of why such high concentrations of adipocyte- and macrophage-specific FABP4 were found in vitreous specimens derived from patients with PDR remains unknown. However, recent studies suggest that FABP4 may be more widely expressed than previously thought, and in fact, FABP4 is also expressed in capillary and venous, but not arterial, endothelial cells under normal conditions^6,7^. The presence of V-FABP4 is not surprising, and suggest that V-FABP4 is most likely derived from retinal capillaries and venous tissue that is affected by PDR. However, the actual origin of the V-FABP4 remains speculative. In addition, a previous study indicated that FABP4 is known to be an indicative biomarker of a general inflammatory degenerative or disease state associated with several metabolic and cardiovascular diseases^8–15^. Therefore, to elucidate origin of the V-FABP4 and their possible pathophysiological roles, further study using suitable animal models will be required to detect what kinds of inflammatory, immune and other cellular signaling are induced.

In terms of relationships between FABP4 and VEGFA, it was reported that VEGFA via VEGF receptor 2 or basic fibroblast growth factor (bFGF) induces the expression of FABP4 in endothelial cells, and in turn, FABP4 in endothelial cells promotes angiogenesis^27^. Such an effect of VEGFA on FABP4 expression was inhibited by chemical inhibition or the short-hairpin (sh) RNA-mediated knockdown of the VEGF-receptor-2 (R2), whereas the VEGFR1 agonists, placental growth factors 1 and 2 had no effect on the expression of FABP4^20^. In addition, the knockdown of FABP4 in endothelial cells significantly reduced their proliferation both under baseline conditions and in response to VEGF and bFGF^20^. Alternatively, unlike VEGF, it was reported that the expression of FABP4 in microvascular endothelial cells is induced by cellular senescence and oxidative stress^28^, and is ectopically induced in injured arterial endothelial cells^29^. Since, as above, FABP4 is expressed not only in adipocytes and macrophages but also in several other types of tissues and cells under physiological and pathophysiological conditions, FABP4 may contribute to several aspects of metabolic and cardiovascular diseases as well as renal, respiratory, neurological, gynecological and oncological diseases^8,15^. On the other hand, several drugs, including a statin^30^, eicosatetraenoic acid (EPA)/docosahexaenoic acid (DHA) agent^31^, dipeptidyl peptidase 4 inhibitor (DPP4i)^32^ and angiotensin II receptor blocker (ARB)^33^ could decrease FABP4 levels.

It is known that angiotensin II (AT II) and components of the renin-angiotensin system (RAS) are expressed in the retina^34^. AT II promotes retinal leukostasis by activating the angiotensin type 1 receptor (AT1-R) pathway that stimulates proinflammatory, proliferative mediators, thus leading to the development and progression of PDR^35^ and choroidal neovascularization (CNV)^36^. Selectively blocking the AT1-R, angiotensin receptor blockers (ARBs) has been shown to have neuroprotective and anti-inflammatory effects in animal models with retinal angiogenesis and neovascularization^37–39^. In fact, several clinical trials have revealed that inhibiting the RAS by ARB successfully suppressed the incidence and progression of DR^40^. Given the observation that ARB could decrease FABP4 expression as describes above, we rationally speculate that a mechanism involving FABP4 may also contribute to such ARB induced beneficial effects toward DR.

To our knowledge, this is the first study to document the presence of V-FABP4 in patients with PDR. However, the current study has several limitations that need to be considered; First, the numbers of patients enrolled in the study were relatively small (n = 40). Nevertheless, in spite of such small numbers in the study groups, we observed a quite strong correlation between V-FABP4 and V-VEGFA (r = 0.72, P < 0.001). Furthermore, elevation of V-VEGFA levels is the consensus observation based on a number of previous studies^41^. Second, current several statistical analyses strongly suggested that V-FABP4 may be involved in the pathogenesis of PDR. However, the precise mechanisms responsible for the pathological contribution of V-FABP4 remains to be elucidated. Therefore, further investigations of the source of the relationship between V-FABP4, V-VEGFA and other related factors within the pathogenesis using larger numbers of patients with PDR with different stages of the disease will also be needed, in addition to in vitro and in vivo studies using animal models as above.
